# Circadian Rhythm of Salivary Cortisol in Obese Adolescents With and Without Apnea: A Pilot Study

**DOI:** 10.3389/fped.2022.795635

**Published:** 2022-04-26

**Authors:** Olga Berdina, Irina Madaeva, Svetlana Bolshakova, Leonid Sholokhov, Liubov Rychkova

**Affiliations:** ^1^Laboratory of Somnology and Neurophysiology, Department of Personalized and Preventive Medicine, Scientific Centre for Family Health and Human Reproduction Problems, Irkutsk, Russia; ^2^Laboratory of Physiology and Pathology of Endocrine System, Department of Reproductive Health Care, Scientific Centre for Family Health and Human Reproduction Problems, Irkutsk, Russia; ^3^Scientific Centre for Family Health and Human Reproduction Problems, Irkutsk, Russia

**Keywords:** salivary cortisol, circadian rhythm, obstructive sleep apnea, obesity, adolescents

## Abstract

**Background and Objective:**

Obstructive sleep apnea (OSA) and obesity are associated with stress system activation involving the hypothalamic-pituitary-adrenal (HPA) axis in adults, but these effects in childhood and adolescence remain unclear. We examined diurnal salivary cortisol as a measurement of the HPA axis function in obese adolescents with and without OSA and the relationships between cortisol levels, body weight, and parameters of polysomnography (PSG).

**Methods:**

After PSG, saliva samples were collected from obese participants (with and without OSA) and lean participants four times over a 24-h period, namely, at 7:00 h (m-sCort), 13:00 h (a-sCort), 19:00 h (e-sCort), and 23:00 h (n-sCort). An enzyme-linked immunosorbent assay (ELISA) was used to measure salivary cortisol levels. The mean values of cortisol levels and fixed-time point diurnal cortisol slope (DCS) were calculated and compared among the three study groups. Correlations between parameters were analyzed using Spearman's correlation coefficients.

**Results:**

Obese OSA participants had significantly higher e-sCort and n-sCort levels than both obese non-OSA participants and lean controls. However, m-sCort and a-sCort in these patients had a pronounced upward trend. M-sCort was significantly correlated with both the lowest oxygen saturation (SpO_2_) and time with SpO_2_ <90%. Moreover, in the obese OSA group, DCS was significantly flatter than in the other two groups. The a-sCort in obese non-OSA participants was significantly higher than that in the lean control group and, surprisingly, was positively correlated with the apnea/hypopnea index. Additionally, m-sCort was related to body weight.

**Conclusion:**

This study provided further evidence for alterations in diurnal cortisol production in obese adolescents, which may indicate a chronically stressed HPA axis. However, there were significant differences in salivary cortisol parameters between participants with and without OSA. Furthermore, patients with OSA had more associations between time-point cortisol levels and OSA-related indices. Nonetheless, this research is a pilot study, and further investigations are necessary.

## Introduction

Sleep is an important part of our lives. It is essential for all functions of an organism (i.e., cellular, organic, or systemic). Altered sleep is potentially harmful to human health and may result in perturbations in cardiovascular, metabolic, neurocognitive, immune, and endocrine functions. It is known that diurnal changes in human physiology, including hormone production, are driven by the endogenous circadian timing system (ECTS), which is composed of the suprachiasmatic nucleus of the hypothalamus and circadian oscillators in peripheral tissues. ECTS enables a consolidated period of wakefulness during the day as well as consolidated sleep during the night (1). Under adequate conditions, ECTS and the sleep-wake cycle (SWC) are synchronized and appropriately regulate the levels of numerous hormones. However, when the SWC and ECTS are desynchronized (e.g., during shift work or in individuals with sleep disorders), normal circadian variations of hormones are altered, which may have adverse health consequences. Among the hormonal consequences of insufficient sleep, there are reductions in testosterone, ovarian hormones, insulin-like growth factor 1, growth hormone, and an increase in cortisol secretion (a relative state of hypercortisolism in the evening hours or a slower decline of cortisol levels across the day) (2–4). There are several hypotheses regarding cortisol circadian rhythm alterations including a slower rate of recovery of the hypothalamic-pituitary-adrenal (HPA) axis from the cortisol awakening response (5). Furthermore, a direct stimulatory effect of sleep loss on the HPA activity can occur (6), and under chronic conditions, elevated cortisol levels in the evening are likely to disturb sleep (3).

Notably, cortisol is an important neuroendocrine biomarker, which is present in all bodily fluids, including urine, serum, and saliva. Although measurements of serum cortisol have traditionally been used as an indicator of the HPA axis activity in clinical and scientific studies, even a small amount of stress, such as venipuncture, can increase hormone levels (7). Moreover, serial blood sampling during wakefulness or nocturnal sleep is not practical. Urinary measurements cannot reflect rapid diurnal changes in cortisol levels, and serial urinary sampling may be inconvenient for patients. In contrast, salivary sampling is a non-invasive and easy method that is more comfortable for patients and suitable for the measurement of diurnal cortisol (and some other biomarkers) in children and adolescents (8, 9).

Obstructive sleep apnea (OSA) and obesity are associated with the activation of the HPA axis. OSA is a common sleep disorder characterized by snoring, obstruction of the upper airways during sleep, intermittent nocturnal hypoxia, and sleep fragmentation, resulting in significant health consequences. OSA affects at least 2% of adolescents globally (10). Among patients with OSA, higher cortisol levels are associated with obesity, which itself is an HPA axis modulator (11–13). The available research on the association between OSA and cortisol levels has focused mainly on obese middle-aged male populations. However, childhood obesity has had an estimated 10-fold increase over the past 40 years globally (14) and is considered the strongest risk factor for OSA and metabolic complications (mainly in adolescence) (15). Some studies have found changes in cortisol levels in children with OSA (8, 16–18); however, current evidence does not provide clear indications as to whether associations exist between OSA in adolescents, obesity, and HPA axis activity.

The aim of our study was to evaluate diurnal salivary cortisol as a measure of the HPA axis activity, in obese adolescents with and without OSA, and determine the relationships between cortisol levels, body weight, and the parameters of polysomnography (PSG). We hypothesized that the circadian rhythm of salivary cortisol is different in these patients. Furthermore, we expected that obese adolescents with OSA would show HPA axis hyperactivity and have more significant correlations between PSG indices and circadian cortisol levels compared with those of obese patients without OSA.

## Materials and Methods

### Study Design and Subjects

In this study, we recruited adolescents who were referred to the Clinic of the Scientific Center for Family Health and Human Reproduction Problems between September 2018 and December 2020 for overweight and obesity management and normal-weight age-matched controls from the community. We screened 99 adolescents (72 boys with obesity and 27 lean peers) against the inclusion and exclusion criteria and enrolled 84 participants (63 obese boys and 21 lean boys) in this cross-sectional study. Of those, four adolescents were lost at follow-up. Our final sample for the future study and analysis included 80 adolescents comprised of 60 obese adolescents and 20 lean control participants.

Eligible participants met the following criteria: male sex, age 15–17 years, body mass index (BMI) ≥95th percentile for obese patients and BMI in the range between 5th and 85th percentile for lean participants, no intake of sleep-promoting pills, the performance of usual activities, no stress for at least 1 week before the measurement period, and signed informed consent. Exclusion criteria included neuromuscular diseases and craniofacial anomalies, positive airway pressure therapy (PAP), and unwillingness to participate in the study. Basic characteristics were obtained for all subjects, and a clinical examination was performed.

The participants completed the Adolescent Sleep Habits Survey, which was adapted for Russian schoolchildren (19), to assess their habitual sleep as well as its disturbances (including OSA symptoms). The questionnaire included questions on sleep, sleep/wake rhythms, breathing during sleep, daytime sleepiness, hypnotic intake history, and previous surgeries (e.g., adenotonsillectomy).

All subjects completed the salivary cortisol portion and PSG of this study. The assignment of obese subjects to subgroups was based on the PSG results: 33 adolescents were included in the OSA obese group and 27 adolescents without OSA were age-matched as obese controls. OSA was identified if the apnea/hypopnea index (AHI) was ≥2 number/h (20). All 20 lean participants also continued to take part in the study after PSG.

### Data Collection and Determination

#### Anthropometric Measurements

Height was measured in centimeters with a stadiometer to the nearest 0.1 cm and converted to meters. Weight was measured in kilograms on a calibrated scale to the nearest 0.1 kg. BMI was calculated as weight in kilograms over height in meters squared (kg/m^**2**^). BMI-for-age percentiles were computed using the Centers for Disease Control and Prevention (CDC) reference values (2–20 years) (21). Obesity was identified if the BMI was more than or equal to the 95th percentile; normal weight was identified if BMI was in the range from 5th to 85th percentile. BMI standard deviation scores (BMI-SDS) were calculated using the least mean squares method.

#### Polysomnography and Sleep Data Detection

The standard overnight PSG was performed at the sleep center. Recordings were commencing in the period of time between 21:00 and 22:00 h and ending at about 06:30 h. The following parameters were recorded using The Grass Comet PSG System (GRASS-TELEFACTOR, USA): electroencephalogram, oculogram, submental and tibial electromyogram, electrocardiogram, chest and abdominal respiratory movements, oronasal airflow, body position, and blood oxygen saturation (SpO_2_). Sleep stages and associated events were scored according to the American Academy of Sleep Medicine (AASM) scoring rules (22). Sleep macrostructure parameters were determined, including total sleep time (TST), non-rapid eye movement sleep stages 1 and 2 (N1–N2), slow-wave sleep (SWS), and rapid eye movement sleep (REM) stages. Cortical respiratory arousals were defined as those occurring within 3 s following an apnea, hypopnea, or snore and expressed as the total number of events per hour of sleep (respiratory arousal index, RAI). The snore index was determined as the number of snore events per hour of sleep. Obstructive hypopneas were defined as events with a drop of ≥30% and apneas with a drop of ≥90% peak respiratory signal amplitude lasting for ≥10 s, which is associated with 3% oxygen desaturation and/or arousal. Obstructive AHI was calculated as the mean number of obstructive apneas and hypopneas per hour of sleep in agreement with the AASM Manual for the scoring of sleep and associated events (22). A certified sleep technologist with extensive experience performed PSG scoring, and a somnologist reviewed the sleep studies.

#### Saliva Collection

One day prior to PSG, after the adolescents were enrolled in the study, saliva samples for measuring cortisol levels were collected from each participant four times over a 24-h period, namely, at 7:00 h (within 30 min of waking up), 13:00, 19:00, and 23:00 h [the author's technique for saliva collection was developed and applied by Madaeva et al. (23) and tested on adolescents (9)]. A sampling of saliva (4–5 ml) collected within 30 min after eating was avoided, and adolescents were told not to eat or drink milk products or sugary foods on the day of collection. Then, 10 min before saliva sampling, the participants rinsed their mouths with water. To collect saliva samples, special polypropylene tubes (SaliCaps, IBL International GmbH, Hamburg, Germany) were used, on which the subject's code and time of collection were indicated. The samples were then refrigerated within 30 min and frozen at or below 20°C within 4 h of collection.

#### Salivary Cortisol Determination

On the day of the assay, samples were completely thawed at room temperature, vortexed, and centrifuged at (3,000 rpm) for 15 min in a local hormonal laboratory. Cortisol levels (ng/ml) in saliva samples were measured by enzyme-linked immunosorbent assay (ELISA), using a commercial Direct Saliva Cortisol ELISA Kit (DBC, Canada) on an ELx808™ Absorbance Microplate Reader (BioTek Instruments, Inc., VT, USA) with a filter set at 450 nm and an upper optical density (OD) limit of 3.0. The lower detection limit was calculated from the standard curve by determining the resulting concentration of the mean OD of the calibrator (based on 10 replicate analyses) minus 2 SD. The kit sensitivity was 1.0 ng/ml; cross-reactivity was 100%.

### Data Analysis

Statistical analysis was performed using Statistica for Windows version 10.0 (StatSoft Inc., USA). The normality of the data was evaluated using the Shapiro–Wilk test. Normally distributed variables are expressed as mean ± standard deviation (M ± SD). Non-normally distributed variables were expressed as medians with interquartile ranges [Me (25%; 75%)]. Continuous variables comparisons between the obese OSA group, obese controls, and lean controls were performed using analysis of variance (ANOVA) or the non-parametric Mann–Whitney (M–W) *U*-test. The Pearson χ^2^ test was used to compare adolescents based on the availability of OSA symptoms (yes/no). Correlations between parameters were analyzed using Spearman's rank correlation coefficients (*R*_s_). The level of statistical significance was set at *P* < 0.05.

### Compliance With Ethical Standards

All procedures performed in this study involving human participants were in accordance with the ethical standards of the national research committee and with the 1964 Helsinki Declaration and its later amendments. This study was reviewed and approved by the Committee on Biomedical Ethics of the Scientific Center for Family Health and Human Reproduction Problems (Protocol No. 2, 23/02/2018). All adolescents and their parents or legal guardians provided written informed consent to participate in this study.

## Results

### Participant's Characteristics and Polysomnographic Data

The baseline characteristics of the study participants are shown in Table 1. The comparative analysis of anthropometric characteristics showed that obese adolescents with OSA did not significantly differ from obese adolescents without OSA and lean controls in age. As expected, BMI and BMI percentiles significantly differed in lean participants compared with those in both obese groups (*p* < 0.0001 and 0.003, respectively) but did not differ between obese groups (the obese with OSA group and obese controls). According to the sleep self-assessment, OSA symptoms had a significant proportion of obese respondents with OSA than obese without OSA adolescents and more than the lean control group (there were two persons with daytime sleepiness). Slightly more than half of the obese respondents with OSA snored. Obese respondents without OSA also snored, but significantly less than the obese participants with OSA (51.5 vs. 11.1%, *p* < 0.0001). Lean adolescents were self-confessed “nonsnorers.” Breathing pauses during sleep were self-reported by only 11 apneic subjects. There was a significant difference between the obese with OSA group and obese controls (45.4 vs. 14.8%, *p* = 0.003), as well as between obese participants and lean controls (45.4 vs. 10%, *p* = 0.001) with respect to daytime sleepiness.

**Table 1:** Baseline study group's characteristics.

**Parameters**	**Obese with** **OSA group** **(*n* = 33)**	**Obese without** **OSA group** **(*n* = 27)**	**Lean control** **group** **(*n* = 20)**	***p*-value^a^**	***p*-value^b^**	***p*-value^c^**	***p*-value^d^**
Age, years	16.3 ± 0.1	16.2± 0.4	16.1 ± 0.3	0.547	0.671	0.574	0.726
BMI-SDS	2.5 ± 0.4	2.4 ± 0.2	−0.08 ± 0.1	**<0.0001**	0.753	**0.0000**	**<0.0001**
BMI, ‰	97.1 ± 1.1	96.3 ± 1.2	67.4 ± 6.8	**0.003**	0.675	**0.0001**	**0.0002**
OSA symptoms, *N* (%)							
Snore	17 (51.5)	3 (11.1)	0 (0.0)	**<0.0001**	**<0.0001**	–	–
Sleep breathing pauses	11 (33.3)	0 (0.0)	0 (0.0)	–	–	–	–
Daytime sleepiness	15 (45.4)	4 (14.8)	2 (10)	**0.004**	**0.003**	**0.001**	0.312

*Data are presented as mean with standard deviation (M ± SD) or number and percentage [N (%)]*.

*OSA, obstructive sleep apnea; BMI-SDS, body mass index, standard deviation score*.

a *p-value of difference between the obese with OSA group and the obese without OSA group and the lean control group*.

b *p-value of difference between the obese with OSA group and the obese without OSA group*.

c *p-value of difference between the obese with OSA group and the lean control group*.

d *p-value of difference between the obese without OSA group and the lean control group*.

*Bold values represent significant findings at p < 0.05*.

Polysomnography data of the three groups are summarized in Table 2. Sleep architecture revealed significant differences between the 1st and the 2nd groups, as well as between those and lean individuals. Therefore, the obese with OSA group had a higher mean snore index (*p* = 0.001), lower average and minimal SpO_2_ (*p* = 0.02 and *p* = 0.01, accordingly), higher RAI (*p* = 0.003), more time spent in superficial sleep (*p* = 0.01), and less time spent in both deep (*p* = 0.06), and REM sleep (*p* = 0.03) than the obese without OSA group and lean controls. Almost all PSG parameters in the obese non-OSA and lean controls were not significantly different, except for the snore and the number of respiratory arousals (*p* = 0.002 and *p* = 0.006, respectively).

**Table 2:** Polysomnographic data of the three groups of participants.

**Parameters**	**Obese with** **OSA group** **(*n* = 33)**	**Obese without** **OSA group** **(*n* = 27)**	**Lean control** **group** **(*n* = 20)**	***p*-value^a^**	***p*-value^b^**	***p*-value^c^**	***p*-value^d^**
Snore index, n/h	74.3 (25.8; 98.5)	13.1 (4.9; 16;2)	1.9 (0.2; 1.6)	**0.001**	**<0.001**	**<0.0001**	**0.002**
AHI, n/h	15.3 (10.4;17.3)	1.2 (0.4;1.7)	1.1(0.3; 1.6)	**0.001**	**<0.0001**	**<0.0001**	0.824
Average SpO2, %	91.3 ± 1.2	96.6 ± 0.7	97.1 ± 0.5	**0.02**	**0.015**	**0.003**	0.765
Lowest SpO2, %	83.7 ± 2.1	94.9 ± 0.5	96.0 ± 0.3	**0.01**	**<0.001**	**<0.001**	0.265
Respiratory arousal index, n/h	26.7 ± 3.2	12.1 ± 1.7	0.5 ± 0.2	**0.003**	**0.002**	**<0.0001**	**0.006**
TST, min	427.1 ± 31.3	429.2 ± 34.1	421 ± 38.2	0.478	0.432	0.184	0.112
N1-N2, % TST	75.1 ± 9.5	56.1 ± 6.2	55.1 ± 4.1	**0.01**	**<0.001**	**<0.001**	0.821
SWS, % TST	14.4 ± 1.3	22.1 ± 2.2	23.4 ± 2.1	0.06	**0.004**	**0.002**	0.823
REM, % TST	13.1 ± 3.3	21.4 ± 3.6	22.3 ± 2.6	**0.03**	**0.003**	**0.002**	0.789

*Data are presented as mean with standard deviation (M ± SD) or median and the 1st quartile; the 3rd quartile [Me (25%; 75%)]*.

*OSA, obstructive sleep apnea; AHI, apnea-hypopnea index; SpO_2_, oxyhemoglobin saturation; TST, total sleep time; N1 and N2, sleep stages 1 and 2; SWS, slow-wave sleep; REM, rapid eye movement sleep*.

a *p-value of difference between the obese with OSA group and the obese without OSA group and the lean control group*.

b *p-value of difference between the obese with OSA group and the obese without OSA group*.

c *p-value of difference between the obese with OSA group and the lean control group*.

d *p-value of difference between the obese without OSA group and the lean control group*.

*Bold values represent significant findings at p < 0.05*.

### Salivary Cortisol Levels

**Figure 1:**
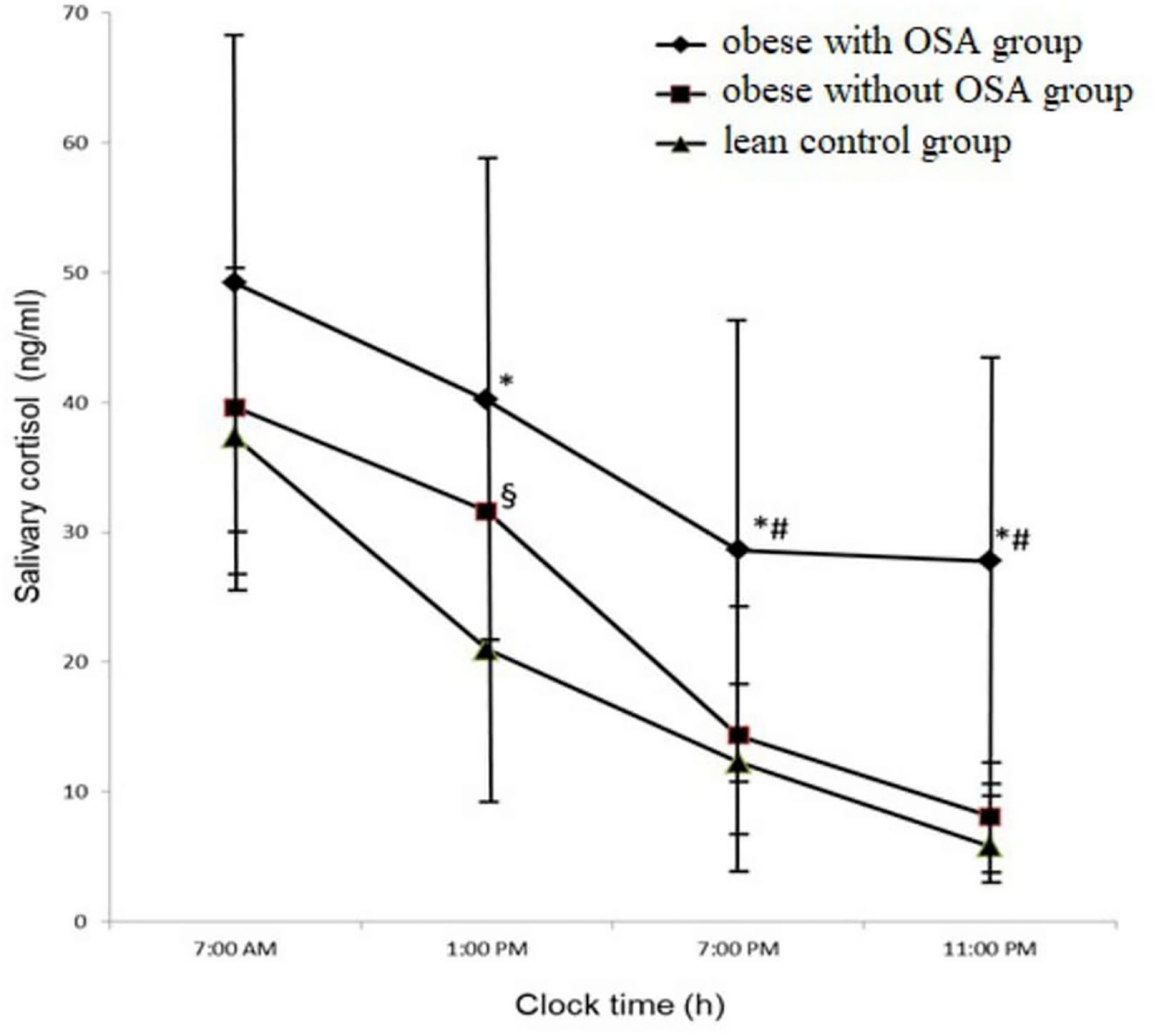
The circadian trajectories of salivary cortisol in the study population. Results were expressed as mean with standard deviation (M ± SD). *p < 0.05 for the obese with OSA group value vs. the lean control group value; ^#^*p* < 0.05 for the obese with OSA group value vs. the obese without OSA group value; ^§^*p* < 0.05 for the obese without OSA group value vs. the lean control group value. OSA, Obstructive sleep apnea.

As shown in Figure 1, salivary cortisol levels were measured at four time points during 24 h in the morning (i.e., m-sCort), afternoon (i.e., a-sCort), evening (i.e., e-sCort), and night (i.e., n-sCort). Surprisingly, in the obese with OSA group, m-sCort was similar to that in the obese control group and the lean control group (49.21 ± 19.22 ng/ml vs. 39.62 ± 12.06 ng/ml vs. 37.32 ± 12.47 ng/ml, *p* = 0.215, respectively), although this measurement had a pronounced upward trend. However, e-sCort and n-sCort in obese with OSA participants were significantly higher compared with those in the lean control group (28.65 ± 18.67 ng/ml vs. 12.31 ± 5.66 ng/ml, *p* = 0.003 and 27.78 ± 18.37 ng/ml vs. 5.8 ± 3.066 ng/ml, *p* = 0.000, respectively) as well as to those with obesity but without OSA (14.35 ± 10.54 ng/ml, *p* = 0.015 and 8.09 ± 4.42 ng/ml, *p* = 0.005, respectively). There were also significant differences between a-sCort levels in the obese with OSA group and the lean control group (40.19 ± 17.89 ng/ml vs. 20.97 ± 12.03 ng/ml, *p* = 0.007). The a-sCort in obese non-OSA participants was significantly higher than that in lean controls (33.12 ± 9.76 ng/ml vs. 20.97 ± 12.03 ng/ml, *p* = 0.032) but was similar to that in the obese with OSA group.

An interesting component of the circadian rhythm of cortisol is the diurnal cortisol slope (DCS). This measurement is the degree of change in cortisol concentrations from morning to evening during wakefulness. In our study, we calculated fixed-time point slopes, in which samples were gathered (24). We used difference scores from morning to night cortisol levels. In the obese with OSA group, the DCS was significantly flatter than in the two other groups (the obese non-OSA group and the lean control group) (21.42 ± 1.15 ng/ml vs. 31.53 ± 8.175 ng/ml and 31.43 ± 6.67 ng/ml, with *p* = 031, respectively), which is clearly identified in Figure 1.

### Correlates of Salivary Cortisol Levels With BMI and Sleep Parameters

Spearman's rank correlation analysis was performed to assess correlations of m-sCort, a-sCor, e-sCort, and n-sCort levels with BMI and sleep indices in obese adolescents with and without OSA. The results are shown in Table 3.

**Table 3:** Cortisol level correlations both with body mass index (BMI) and polysomnography indices in obese adolescents with/without obstructive sleep apnea (OSA).

**Parameter**	**m-sCort**		**a-sCort**		**e-sCort**		**n-sCort**	
	***R*_S_**	***P*-value**	***R*_S_**	***P*-value**	***R*_S_**	***P*-value**	***R*_S_**	***P*-value**
BMI-SDS	0.264**/** **0.476**	0.406/ **0.046**	0.195/ 0.301	0.543/ 0.226	−0.129/ 0.369	0.687/ 0.132	0.209/ −0.284	0.515/ 0.254
AHI	0.224/ −0.160	0.484/ 0.989	0.307/ **0.564**	0.332/ **0.015**	0.376/ 0.276	0.228/ 0.267	**0.499**/ −0.111	**0.039**/ 0.660
Lowest SpO_2_	**−0.572**/ 0.383	**0.042/** 0.117	−0.345/ −0.067	0.272/ 0.792	−0.087/ 0.371	0.789**/** 0.130	−0.373/ −0.092	0.233/ 0.718
Time with SpO_2_ <90%	**0.648**/ −0.132	**0.023/** 0.602	−0.098/ −0.256	0761/ 0.305	−0.519/ −0.441	0.084**/** 0.067	0.226/ −0.096	0.480/ 0.703
RAI	−0.225/ −0.308	0.482/ 0.213	−0.525/ −0.348	0.079/ 0.891	−0.303/ −0.084	0.339/ 0.740	−0.513/ −0.168	0.075/ 0.506
TST	−0.290/ 0.084	0.360/ 0.741	−0.406/ −0.002	0.190/ 0.993	−0.493/ −0.083	0.103/ 0.744	−0.288/ −0.045	0.364/ 0.860
N1-N2	0.166/ 0.252	0.605/ 0.314	0.538/ −0.351	0.071/ 0.154	0.241/ −0.341	0.451/ 0.165	**0.764**/ 0.207	**0.004**/ 0.411
SWS	0.213/ 0.398	0.507/ 0.891	−0.427/ −0.159	0.166/ 0.528	**−0.737**/ 0.053	**0.006**/ 0.834	−0.389/ −0.321	0.212/ 0.072/
REM	−0.340/ −0.321	0.279/ 0.195	−0.113/ −0.080	0.726/ 0.752	−0.235/ −0.096	0.462/ 0.704	−0.540/ 0.007	0.070/ 0.979

*Data are presented as Spearman's rank correlation coefficients (R_s_) and p-values*.

*BMI-SDS, body mass index, standard deviation score; OSA, obstructive sleep apnea; AHI, apnea-hypopnea index; SpO_2_, oxyhemoglobin saturation; RAI, respiratory arousal index; TST, total sleep time; N1 and N2, sleep stages 1 and 2; SWS, slow-wave sleep; REM, rapid eye movement sleep*.

*Bold values represent significant findings at p < 0.05*.

Salivary morning cortisol was found to correlate significantly with BMI-SDS (*R*_s_ =0.476; *p* = 0.046) in obese non-OSA participants, and with the lowest SpO_2_ (*R*_s_ = −0.572; *p* = 0.042), and time with SpO_2_ <90% (*R*_s_ = 0.648; *p* = 0.023) in obese patients with OSA. Interestingly, a-sCort was significantly positively correlated with AHI in the obese non-OSA group. Of note, salivary evening cortisol, as well as night cortisol, levels were significantly correlated with PSG parameters such as SWS (*R*_s_ = −0.737; *p* = 0.006 for e-sCort) for both AHI and N1–N2 (*R*_s_ = 0.499; *p* = 0.039 and *R*_s_ = 0.764; *p* = 0.004, respectively, for n-sCort) in obese OSA adolescents. Finally, circadian salivary cortisol levels did not correlate significantly with the other studied PSG characteristics such as RAI, TST, and REM sleep in any of the groups.

## Discussion

This study provides further evidence for alterations in circadian HPA activity in obese adolescents, which may indicate a chronically stressed HPA axis. However, there were significant differences in salivary cortisol parameters between participants with OSA and without OSA. These changes affect salivary cortisol levels depending on the time of day. It is known that OSA (25) and obesity (26) may affect HPA, due to hypoxic episodes and sleep fragmentation, stresses, and metabolic dysregulation. Salivary cortisol, an important biomarker of HPA activity, is a reliable surrogate for free, biologically active cortisol in the plasma and is especially important in pediatric studies. Some studies from the last two decades have reported controversial data regarding associations between the features of salivary or plasma diurnal cortisol rhythmicity both in obese adults and children with OSA, as well as before and after treatment for OSA. So, Dadoun et al. (13) showed no association between OSA and changes in salivary and plasma cortisol levels or the features of their diurnal rhythms. Furthermore, they proved that men with obesity have lower plasma cortisol levels than normal-weight subjects. Only men were included in this study and other studies, potentially due to a higher prevalence of OSA in obese men. In this study, we recruited only male participants but did not obtain similar results. Raff et al. (27) evaluated the effect of PAP therapy on salivary cortisol concentration as an index of stress related to sleepiness and OSA. In this study, saliva samples were obtained at two time points (23:00 and 7:00 h). It should be noted that these points of saliva collection coincided with two out of the four time points. Notably, no differences between patients with OSA and controls before starting treatment and no significant correlation between OSA severity and either morning or evening salivary cortisol levels. Similar results were described in a meta-analysis by Imani et al. (12), who observed no significant differences in serum cortisol levels, plasma cortisol levels, and morning salivary cortisol levels between OSA and non-OSA adults. There were interesting findings of stronger HPA axis activation in non-obese men with OSA compared with obese patients with OSA (28). All of these studies' results among adult patients are in contrast with our findings in obese adolescents with and without OSA. However, similar data were obtained. Vgontzas et al. (29) and Edwards et al. (30) reported an increased HPA axis activity in patients with OSA compared with healthy controls and a significant association between OSA severity and diurnal cortisol values.

Similar to the results in adult studies, we found highly contradictory data regarding the circadian cortisol rhythm in children and youth. Some characteristics are known to influence cortisol concentrations (e.g., the duration of obesity, obesity-associated metabolic disturbances, sleep disturbances, such as OSA, ENT pathology, and emotional and behavioral problems) and may explain the discrepancies in cortisol levels observed in these studies. Malakasioti et al. (16), Park et al. (17), and Jeong et al. (18) reported that children with OSA aged 2–13 years had lower morning cortisol levels (salivary or serum) than those in the control group but similar cortisol concentrations at night. In contrast, Patacchioli et al. (8) found increased diurnal salivary cortisol production in children with OSA, but salivary cortisol levels in the morning were negatively associated with OSA severity. However, these children were not adolescents and were not overweight or obese but had tonsillar hypertrophy; therefore, these results cannot be extrapolated to our study sample. In contrast, our results showed significant positive correlations between the salivary cortisol level and AHI (n-sCort), as well as with SpO_2_ <90% (m-sCort) but negative correlations with lowest SpO_2_ (m-sCort) in obese OSA youth. These data suggest that circadian cortisol levels can be predictors of OSA severity, and this requires further research.

The attempts to establish a direct connection between obesity/metabolic syndrome (MS) and cortisol excretion brought contradictory results (31, 32). So, Jackson et al. proved that the hair cortisol concentration positively correlated with BMI and waist circumference and statistically significantly increased in obese patients (33). In opposite, Fan et al. reported a negative correlation between morning serum cortisol and different obesity indices in Chinese rural populations (34). There are associations between the level of salivary or urinary cortisol and MS components among elderly patients (35). Andrew et al. concluded that in obesity, the metabolism of cortisol is impaired, and the conversion of cortisone to cortisol by 11 beta-reductase is enhanced (36). These observations suggest that cortisol clearance is altered in obese patients, and this may account for the activation of the HPA axis. Authors suggested that obese subjects will have higher concentrations of cortisol in key target tissues, for example, visceral fat and liver, with adverse consequences for health. These results are reflected in our study on adolescents.

Notably, adolescence (especially, aged 15–17 years) is a time of important physical, hormonal, and psychological changes (37), and daily stressful situations may have adverse effects on the regulation of the HPA axis activity, sleep quality, and weight status. However, although the role of stress and HPA axis dysfunction in the development of MS or obesity in adults without OSA has been widely discussed, in adolescents, this evidence remains inconclusive and unclear (38, 39). Similar to our results, previously reported elevated cortisol levels in obese youth (aged 12–19 years) (40) and in obese children and adolescents (aged 2–18 years) with OSA (41), but studies did not find this association (38, 42). Interestingly, Wirix et al. (43) and Yu et al. (44) reported an increased cortisol production rate and lower morning and evening cortisol levels in overweight and obese children, which is opposite to our findings. Martens et al. (45) found elevated morning serum cortisol levels in 20% of overweight or obese children and adolescents, which were associated with higher fasting glucose but showed no association between BMI and morning cortisol concentrations, which contradicts our results. In our study, we also observed a tendency of increased salivary cortisol concentration in the morning in both obese patients with OSA- and non-OSA. However, we also found a positive correlation between BMI-SDS and m-sCort in obese subjects without OSA, confirming the results of some studies in the youth population (46). However, Strait et al. (47) found no correlation between salivary cortisol levels and markers of MS in overweight children. In our study, we also did not find significant differences between obese and non-obese adolescents. Nonetheless, there was a tendency toward higher salivary cortisol levels at all four time points in obese subjects compared with those in lean controls.

It is known that flattened slopes of cortisol secretion, which exhibits suppressed morning peak levels or failures to reach sufficiently low levels by evening, are indicative of the HPA axis dysregulation (48). They may be associated with a higher risk of obesity, hypertension, or type 2 diabetes (32). We found flatter DCS from m-sCort to n-sCort in the obese OSA group and less slope cortisol secretion from m-sCort to a-sCort in obese non-OSA participants. These results can be explained by the predominant influence of nocturnal hypoxemia and fragmented sleep in obese patients with OSA, as well as the influence of day stress in obese patients on the circadian HPA axis activity.

The data from the pilot study provided more pronounced diurnal cortisol alternations in apneic obese male adolescents than in those without OSA. Furthermore, patients with OSA had more associations between time-point cortisol levels and OSA-related indices. Our preliminary findings are consistent with previous reports of HPA axis hyperactivity in adolescents with OSA. Reported modulations of circadian cortisol rhythmicity associated with OSA and obesity may underlie some of the above-mentioned negative health outcomes. At present, evaluation of circadian secretion of free cortisol appears to be promising for the detection of subtle alterations of the HPA axis in conditions, such as obesity and OSA, in daily clinical practice, whereas dynamic tests following stimulation with different substances and psychological stress challenges or suppression with inhibiting agents of the HPA axis at different levels requires the use of specific clinical settings and special patient preparation. Salivary cortisol offers a suitable and easily obtainable measure for assessing diurnal cortisol alternations in children and adolescents and can therefore aid in further advancing understanding in this area, as well as to enhance diagnostic, prognostic, and treatment possibilities in obesity and OSA. Further studies are necessary to elucidate how cortisol metabolism is involved in the pathogenesis of both obesity-induced OSA and *vice versa* in adolescence and to evaluate the effect of PAP therapy on diurnal cortisol levels in this population.

## Limitations

This was a pilot study; therefore, it had several limitations. The major limitation was the small sample size, potentially limiting our power to uncover more associations and predictors in the study groups. We believed that increasing the sample size in our future research will allow us to strengthen the study because we can divide participants according to both OSA and obesity severity and consider a general linear model to evaluate the possible effect of OSA severity, as well as obesity severity, and the comorbidity of OSA with obesity on circadian cortisol concentration. A second limitation of this study was that only men were assessed, which eliminated the gender effect, but the study results can only be applied to male adolescents, which also requires future research to study the associations between cortisol and OSA, body weight, and sex. The last limitation was using BMI-for-age percentiles but not a full assessment of body composition to classify subjects in the study, potentially increasing classification errors. We can revise the methodology in our future research.

## Data Availability Statement

The raw data supporting the conclusions of this article will be made available by the authors, without undue reservation.

## Ethics Statement

The studies involving human participants were reviewed and approved by Committee on Biomedical Ethics of the Scientific Center for Family Health and Human Reproduction Problems. Written informed consent to participate in this study was provided by the participants and their legal guardian/next of kin.

## Author Contributions

IM and LR: conceptualization and design. IM and LS: methodology. OB and SB: investigation and formal analysis. OB, IM, and LS: data curation. OB: writing original draft. OB, IM, SB, LS, and LR: writing–review and editing. LR: supervision. All authors agree to be accountable for the content of the study.

## Conflict of Interest

The authors declare that the research was conducted in the absence of any commercial or financial relationships that could be construed as a potential conflict of interest.

## Publisher's Note

All claims expressed in this article are solely those of the authors and do not necessarily represent those of their affiliated organizations, or those of the publisher, the editors and the reviewers. Any product that may be evaluated in this article, or claim that may be made by its manufacturer, is not guaranteed or endorsed by the publisher.
